# Delta-radiomics features during radiotherapy improve the prediction of late xerostomia

**DOI:** 10.1038/s41598-019-48184-3

**Published:** 2019-08-28

**Authors:** Lisanne V. van Dijk, Johannes A. Langendijk, Tian-Tian Zhai, Thea A. Vedelaar, Walter Noordzij, Roel J. H. M. Steenbakkers, Nanna M. Sijtsema

**Affiliations:** 1 Department of Radiation Oncology, University of Groningen, University Medical Center Groningen, Groningen, The Netherlands; 2 Department of Radiation Oncology, Cancer Hospital of Shantou University Medical College, Shantou, China; 3 Nuclear Medicine and Molecular Imaging, University of Groningen, University Medical Center Groningen, Groningen, The Netherlands

**Keywords:** Head and neck cancer, Saliva

## Abstract

The response of the major salivary glands, the parotid glands, to radiation dose is patient-specific. This study was designed to investigate whether parotid gland changes seen in weekly CT during treatment, quantified by delta-radiomics features (Δfeatures), could improve the prediction of moderate-to-severe xerostomia at 12 months after radiotherapy (Xer_12m_). Parotid gland Δfeatures were extracted from in total 68 planning and 340 weekly CTs, representing geometric, intensity and texture characteristics. Bootstrapped forward variable selection was performed to identify the best predictors of Xer_12m_. The predictive contribution of the resulting Δfeatures to a pre-treatment reference model, based on contralateral parotid gland mean dose and baseline xerostomia scores (Xer_baseline_) only, was evaluated. Xer_12m_ was reported by 26 (38%) of the 68 patients included. The most predictive Δfeature was the contralateral parotid gland surface change, which was significantly associated with Xer_12m_ for all weeks (p < 0.04), but performed best for week 3 (ΔPG-surface_w3_; p < 0.001). Moreover, ∆PG-surface_w3_ showed a significant predictive contribution in addition to the pre-treatment reference model (likelihood-ratio test; p = 0.003), resulting in a significantly better model performance (AUC_train_ = 0.92; AUC_test_ = 0.93) compared to that of the pre-treatment model (AUC_train_ = 0.82; AUC_test_ = 0.82). These results suggest that mid-treatment parotid gland changes substantially improve the prediction of late radiation-induced xerostomia.

## Introduction

Xerostomia is one of the most frequently reported side effects following radiotherapy of head and neck cancer (HNC) patients and affects patient-reported quality of life^1^. For the prediction of late xerostomia, Normal Tissue Complication Probability (NTCP) models have been developed with pre-treatment dose-volume parameters and baseline complaints as most important predictors^2,3^. However, xerostomia NTCP models based on information during treatment are less explored. Since in-treatment parameters contain information on the patient-specific response to treatment, they may resolve some of the unexplained variability that remains for NTCP models that are based on pre-treatment variables only. These in-treatment parameters could therefore be used to improve the prediction of late xerostomia. Adequate prediction supported by in-treatment data may offer new opportunities to guide treatment adaptation aiming at a further reduction of late radiation-induced side effects.

Several studies have investigated changes of the parotid glands during and after treatment in CT images^4–7^ and have shown a weak to moderate relationship between parotid gland dose and volume change^4,6^. However, knowledge of the relationship between parotid gland changes and patient-reported xerostomia is still limited. Therefore, in our previous study, we investigated the association between late patient-reported xerostomia and parotid gland changes quantified in radiomics features, or also called image biomarkers, extracted from CT images before and 6 weeks after treatment. That study showed that the parotid gland surface reduction (∆PG-surface_6w-postRT_) was strongly associated with the development of xerostomia at 6 and 12 months after radiotherapy^8^.

However, this post-treatment model does not allow for treatment adaptation, as the total prescribed radiation dose has already been administered. Hence, the next step is to investigate parotid gland changes during treatment.

The aim of the current study was to identify quantitative parotid gland changes during treatment that predict the development of late xerostomia. These parotid gland changes were extracted from pre-treatment and weekly CT-images during radiotherapy, from which delta radiomics features (∆features) were quantified, representing differences in intensity, texture and geometric characteristics of the parotid glands.

## Results

### Patients

Moderate-to-severe xerostomia was reported by 26 (38%) of all 68 patients included at 12 months after radiotherapy (22 in the training set and 4 in the test set). At 6 months after radiotherapy, the moderate-to-severe xerostomia reporting rate was 46 (52%) out of a total of 88 patients.

### ∆features selection

For the geometric features, a change of contralateral parotid gland surface (∆PG-surface) was the most frequently selected ∆feature for all weeks predicting Xer_12m_ (see Supplementary Data 4 for frequency plot), except for week 2 where the ∆PG-‘bounding box volume’ frequency was slightly higher. The ∆PG-surface frequency was especially high in week 3 (725 times selected in the 1000 bootstrap samples). In other weeks, the subsequently selected ∆features, ∆‘volume’, ∆‘bounding box volume’ and ∆‘volume times mean intensity’, were highly correlated with ∆PG-surface for all weeks with ρ = 0.79–0.93, ρ = 0.66–0.79 and ρ = 0.82–0.91, respectively.

For the intensity and texture ∆features, no clear selection of ∆features that were most frequently selected for all weeks could be made (see Supplementary Data 4 for frequency plot). Overall, the most frequently selected ∆features on average were: coarseness from the neighbourhood grey tone difference matrix (coarseness), kurtosis and the median intensity (median). The ∆features kurtosis and median were weakly correlated to coarseness (ρ = 0.13, 0.18), but were stronger correlated to each other (ρ = 0.79).

A heatmap of all ∆IBMs and histograms of the most frequently selected ∆IBMs are depicted in Supplementary Data 5 to illustrate the distribution and relation between the ∆IBMs.

### ∆feature: univariable analysis

The univariable analysis also showed that ∆PG-surface was the most significant ∆feature. This geometric ∆feature was significantly associated with Xer_12m_ at all weeks (p < 0.04) but was most significant in week 3 (p < 0.001). This week showed the largest regression coefficient of all weeks (Supplementary Data 6).

For the intensity and texture ∆features, none of the most frequently selected ∆features were significantly associated with Xer_12m_ in any of the weeks (coarseness: p ≥ 0.06; kurtosis ≥0.08; median: p ≥ 0.07) (Supplementary Data 6).

### ∆features, dose and toxicity: multivariable analysis

Since the ∆features showed the best performance in week 3, the multivariable analysis was performed with the selected week 3 ∆features only.

In the training set (56 patients), discrimination of the reference ‘pre-treatment’ model (Xer_baseline_ and PGdose) was good (AUC_train_ = 0.83 (AUC_internal.val._ = 0.82)), yet the geometric ∆feature model with ∆PG-surface_w3_ and Xer_baseline_ performed better (∆feature model 1: AUC_train_ = 0.88 (AUC_internal.val._ = 0.84)) in predicting Xer_12m_ (Table 1). Moreover, the addition of ∆PG-surface_w3_ to the pre-treatment model (likelihood-ratio test, p = 0.003), significantly improved different aspects of performance (∆feature model 2: AUC_train_ = 0.92 (AUC_internal.val._ = 0.89); Table 1). Validated in the test set (14 patients), the models showed stable performance (Table 1). Furthermore, also in the test set ∆feature model 2 showed the highest performance (AUC_test_ = 0.93 and R^2^ = 0.49). Ultimately, Table 2 gives the model coefficients that were optimized for the entire cohort, with the coefficients corrected for optimism using bootstrapping. The performance measures of the final model are shown in Supplementary Data 7.

**Table 1:** Performance of NTCP models predicting Xer_12m_ with and without ∆image biomarkers in the training and the test set.

			Pre-treatment reference model	∆feature model 1	∆feature model 2
			Xer_baseline_	Xer_baseline_	Xer_baseline_
			PG dose		PG dose
				∆PG-Surface _w3_	∆PG-Surface _w3_
Training set 56 patients	*Apparent*	Nagelkerke R2	0.46	0.53	0.59
		Area Under the Curve (AUC)	0.83 (0.70–0.96)	0.88 (0.79–0.97)	0.92 (0.85–0.99)
		Discrimination slope	0.39	0.45	0.48
	*Internal validation*	Nagelkerke R2	0.41*	0.45	0.51
		AUC	0.82*	0.84	0.89
Test set 14 patients	*Validation*	Nagelkerke R2	0.36	0.39	0.49
		Area Under the Curve (AUC)	0.80	0.85	0.93

*No variable selection was performed for internal validation of the reference model.

**Table 2:** Estimated coefficients (uncorrected and corrected for optimism) of pre-treatment and ∆image biomarkers models fitted to the entire dataset.

Model		β		odds ratio (95% CI)	p-value
		Uncorrected	Corrected		
Pre-treatment reference model	*intercept*	−3.794	−3.385*		
	Xer_baseline_	2.531	2.280*	12.56 (3.39–46.54)	<0.001
	Parotid gland dose	0.099	0.089*	1.1 (1.03–1.18)	0.005
∆feature model 1	*intercept*	−3.139	−2.515		
	Xer_baseline_	2.533	2.074	12.59 (3.13–50.73)	<0.001
	∆PG-surface_w3_ (cm^2^)	−0.568	−0.465	0.57 (0.41–0.79)	0.001
∆feature model 2	*intercept*	−4.515	−3.305		
	Xer_baseline_	2.591	1.936	13.35 (3.13–56.95)	<0.001
	Parotid gland dose	0.072	0.054	1.07 (0.77–1.51)	0.074
	∆PG-surface_w3_ (cm^2^)	−0.481	−0.360	0.62 (0.57–0.67)	0.005

*No variable selection was performed for internal validation of the reference model.

**Figure 1:**
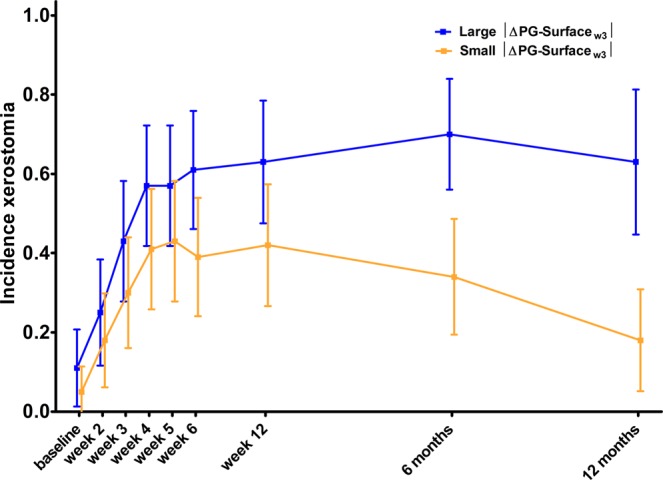
Actual moderate-to-severe xerostomia incidence and 95% confident intervals at baseline, weekly during, and 6 weeks (week 12), 6 months, and 12 months after treatment for patients, with parotid gland surface reduction at week 3 (∆PG-Surface_w3_) larger (blue) or smaller (yellow) than the median reduction (|median| = 2.73).

Acute xerostomia scores at week 3 (Xer_w3_) significantly improved ∆feature model 2 (Xer_baseline_, PGdose and ∆PG-surface_w3_) (likelihood-ratio test, p = 0.01), but the improvement in performance was relatively small (AUC_train_ = 0.93 (AUC_internal.val._ = 0.89)) and decreased when validated in the test set (AUC_test_ = 0.88). The relationship between ∆PG-surface_w3_ and Xer_w3_ was not significant (p = 0.14). This is also demonstrated in Fig. 1, where patients with a large and small surface reduction at week 3 (median ∆PG-surface_w3_ = −2.73) showed a clear differentiation of actual moderate-to-severe xerostomia incidences at 6 or 12 months after treatment, but not for acute time points.

No significant relationship was found between ∆PG-surface and Xer_baseline_ (p = 0.17). Xer_w3_ was significantly associated with both PGdose (p = 0.04) and Xer_baseline_ (p = 0.03), probably explaining the marginal prediction improvement of Xer_3w_ to the ∆feature model with PGdose and Xer_baseline_.

For the secondary endpoint Xer_6m_, ∆PG-surface_w3_ also added significantly to the pre-treatment model in predicting Xer_6m_ (likelihood-ratio test, p = 0.02). See Supplementary Data 8 for more details. None of the frequently selected intensity or texture ∆features showed any significant improvement either compared to or in addition to the pre-treatment model (likelihood-ratio test, p > 0.27) in predicting Xer_12m_ or Xer_6m_.

### Parotid gland dose and ∆features

**Figure 2:**
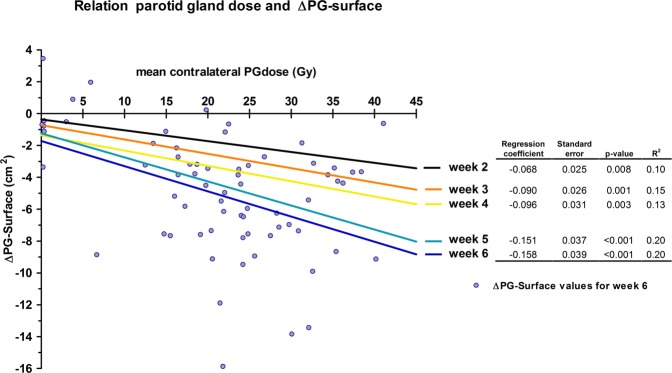
Univariable linear regression of contralateral parotid gland mean dose (PGdose) predicting parotid gland surface reduction (∆PG-surface) for different weeks (lines) and regression characteristics (Table). Correlation increases over time, but remained weak. Data point represent ∆PG-surface values for week 6.

The linear relationship of contralateral parotid gland mean dose and ∆PG-surface was significant for all weeks (p < 0.008; Fig. 2). Depicted in Fig. 2, the regression coefficients of this linear relationship effectively increased over time, as did the coefficient of determination, but remained weak.

The selected intensity and the texture ∆features, ∆median and ∆coarseness were significantly correlated (p < 0.05) to parotid gland dose for week 2, 3, 5, 6 and 5, 6, respectively (Supplementary Data 9). However, the coefficient of determination was relatively low (R^2^ = 0.00–0.21). ∆LZLGE was not significant for any week.

## Discussion

The current study shows that surface change of the contralateral parotid gland (∆PG-surface) assessed during the course of radiotherapy was strongly associated with the development of late xerostomia (Xer_12m_ and Xer_6m_). The association of this geometric ∆feature was statistically significant during the entire course of treatment but performed best for changes obtained between treatment planning and week 3. This time point is still clinically relevant, as any treatment adaptations could still influence the patient’s toxicity outcome. ∆PG-surface_w3_ did not only show improved predictive performance over PGdose, but it also improved the pre-treatment model performance significantly. The resulting model that was based on Xer_baseline_, PGdose and ∆PG-surface_w3_ showed excellent performance when predicting Xer_12m_ in both the train (AUC = 0.92), test (AUC = 0.93) and when fitted to the entire cohort (AUC = 0.91). However, these results should be confirmed by external validation in larger patient cohorts.

Castelli *et al*. showed that parotid gland dose could significantly be reduced with an adaptive radiotherapy approach (ART)^9^. By re-planning the dose distribution on weekly CTs, an average NTCP reduction of 11% (maximum 30%) was observed. However, weekly re-planning is time consuming. This highlights the potential of the ∆feature NTCP model with ∆PG-surface_w3_, since it could select patients during treatment that have a high risk of developing xerostomia. If these high-risk patients could, subsequently, receive less PGdose by re-planning, their risk of xerostomia could be further reduced. Alternatively, the model-based approach has been introduced to select patients for proton therapy. Patients can be selected that have a clinically relevant NTCP-reduction with a proton plan compared to their photon based treatment plan^10^. Proton therapy has the potential to better conform the dose to the tumour while sparing the surrounding normal tissue, due to the intrinsic properties of protons^11^. By incorporating patient-specific ∆feature response information in the pre-treatment reference model, patients that do not initially qualify could be reclassified for proton therapy. Accordingly, treatment can be changed from photon to proton therapy, when relevant differences are seen in the new ΔNTCP values.

In a previous study, the geometric radiomics feature differences were calculated between 6 weeks post-treatment and prior to treatment (∆feature_6week-postRT_)^8^. The association of ∆feature_6week-postRT_ with Xer_12m_ was investigated in a patient cohort (n = 107) independent of the current cohort. Interestingly, the most stable and predictive post-treatment ∆feature was also the contralateral ∆PG-surface. Similar to the results of the current study, inclusion of ∆PG-surface substantially improved the pre-treatment model. Using the same coefficients of the post-treatment model with ∆PG-surface_6w-postRT_, Xer_baseline_ and PGdose in the current cohort, also showed a comparable performance (AUC = 0.89) to that of the model trained in the current cohort (AUC = 0.91). The other way around, using the coefficients of the current model in the previous cohort also resulted in a comparable improvement in performance for the model in the post-treatment cohort (Supplementary Data 10). This suggests that the ∆PG-surface_w3_ model would also perform well when externally validated in a cohort where ∆PG-surface is acquired at week 3. In both studies, ∆PG-volume was highly correlated to ∆PG-surface, and also performed well in predicting late xerostomia.

In line with other studies that observed a relationship between PGdose and PG shrinkage^4,6,12,13^, linear regression in the current study also showed that there was a weak to moderate correlation between ∆PG-surface and PGdose. Interestingly, the correlation between PGdose and ∆PG-surface effectively increased over the time of treatment, illustrated by the increasing values of the regression coefficients and R2 every consecutive week. This suggests that the effect of planning PGdose on ∆PG-surface becomes clearer as more dose is administered. However, such an effect was not seen for the association between ∆PG-surface and Xer_12m_, since the univariable logistic regression coefficient and the performance of ∆PG-surface increased from week 2 to 3, but decreased for the subsequent weeks. Hence, we concluded that the best moment for predicting Xer_12m_ was during week 3. The explanation may be that most parotid glands shrink when irradiated, as reported in previous studies^4–7^, but patients that have a parotid gland that shrinks early in treatment have a higher risk of developing late xerostomia. Therefore, ∆PG-surface_week3_ could be a marker to differentiate between patients that develop permanent damage of the parotid gland versus those that can recover.

In addition to these observations, ∆PG-surface was not associated with acute xerostomia, although it was strongly associated with the development of late xerostomia. Figure 1 also demonstrated this, as ∆PG-surface_w3_ did not show a clear differentiation between the actual incidences of moderate-to-severe xerostomia at week 3 or any of the other acute time points. In contrast, this differentiation can be clearly seen for 6 and 12 months after radiotherapy. Furthermore, acute xerostomia scores at 3 weeks (Xer_w3_) did significantly add to the model with Xer_baseline_, ∆PG-surface and PGdose, although the improvement in performance measures was small. This is probably due to the correlation between Xer_w3_ and both PGdose and Xer_baseline_. Further research needs to be performed on larger datasets in order to investigate whether acute toxicities can contribute to ∆feature models.

Changes in intensity or texture features were not related to the development of xerostomia. In contrast, many of these ∆features were significantly related to PGdose, even though no relationship was seen with the development of xerostomia. Furthermore, detailed investigation of the most frequently selected intensity or texture ∆features showed that these ∆features contained one or two outliers that determined the effect. The influence of outliers indicates the importance of evaluating the selected features before presenting them in a final model. In this study, the analysis of ∆features was used rather than the features directly extracted per week. The results of these absolute weekly features were not significant. In contrast, in a previous study, a pre-treatment CT feature that indicates tissue heterogeneity, was significantly associated with the development of late xerostomia^14^. It might be that the effect of pre-treatment is too weak to be observed in this relatively small dataset. In addition, using proportional ∆features instead of absolute difference ∆features did not improve the results of this study either.

The limitations of this study are the low numbers of patients included in this analysis and no direct external validation was performed. Nevertheless, we have validated our results by splitting our dataset in a training set, for which variables were selected, and tested them in a small unseen cohort. The models performed well in both the training as the test cohort. Finally the models were fitted to the entire dataset, to make full use of the total number of patients in estimating the most optimal coefficients given all data. Furthermore, only pre-treatment PGdose rather than the accumulated dose over all weekly CT scans was evaluated, since this was outside the scope of the paper. Brouwer *et al*. showed that accumulated dose calculated on weekly CTs was almost equal to the pre-treatment PGdose^15^. Using accumulated PGdose could improve the predictive performance of PGdose. Additionally, other modalities, such as positron emission tomography and magnetic resonance imaging could potentially provide better information during treatment on function loss of the PG gland. Future studies using these modalities could improve the quantification and understanding of the development of late xerostomia.

In conclusion, contralateral parotid gland surface area reduction during the course of radiotherapy (ΔPG-surface) was associated with the development of late xerostomia both at 6 and 12 months after radiotherapy. The model consisting of Xer_baseline_, parotid gland dose and ∆PG-surface, as assessed at week 3 during treatment (ΔPG-surface_w3_), showed the best performance, and substantially improved the pre-treatment model based on parotid gland dose and Xer_baseline_ only (from AUC of 0.83 to 0.91). This mid-treatment model may be a good candidate to identify patients most at risk of developing late xerostomia and who may benefit from treatment adaptations, but external validation is warranted.

## Method

### Patients and image acquisition

The study cohort included consecutive HNC patients that were treated with definitive radiotherapy and received weekly CTs between January 2014 and December 2016. The cohort, sorted by treatment start date, was split in a training set (80%; 54 patients) and test set (20%; 14 patients). Radiation plans were adapted where necessary due to anatomical changes causing reduced target coverage. Patients were treated with IMRT or VMAT using a simultaneous integrated boost (SIB) technique, either as a single modality or in combination with concurrent chemotherapy or cetuximab. Plans were optimised to spare the parotid glands and swallowing organs at risk (superior pharyngeal constrictor muscle and supraglottic area) as much as possible without compromising the dose to the target volumes^16^. The primary tumour and pathologic lymph nodes were generally prescribed 70 Gy (2 Gy per fraction) and the cervical lymph node levels were prescribed an elective radiation dose of 54.25 Gy (1.55 Gy per fraction)^17^. More detailed descriptions of the radiation protocols used was reported in previous work^16^. Patient characteristics are listed in Table 3.

**Table 3:** Patient characteristics of patients that had follow-up information available at 12 and 6 months after treatment.

	Follow-up info at 12 months		Follow-up info at 6 months	
Characteristics	N = 68	%	N = 88	%
***Sex***				
Female	20	29	26	30
Male	48	71	62	70
***Age***				
18–65	48	71	62	70
>65	20	29	26	30
***Tumour site***				
Oropharynx	22	32	27	31
Hypopharynx	0	0	1	1
Nasopharynx	5	7	5	6
Larynx	22	32	27	31
Oral cavity	15	22	23	26
Unknown primary	1	1	1	1
Other	3	4	4	5
***Tumour classification***				
T0	1	1	1	1
T1	11	16	14	16
T2	14	21	19	22
T3	17	25	19	22
T4	23	34	33	38
Unknown	2	3	2	2
***Node classification***				
N0	23	34	28	33
N1	9	13	13	15
N2abc	31	46	41	47
N3	3	4	4	5
***Systemic treatment***				
Yes	34	50	47	53
No	34	50	41	47
***Treatment technique***				
IMRT	27	40	30	34
VMAT	41	60	58	66
***Bilateral***				
Yes	57	84	72	82
no	11	16	16	18
***Baseline xerostomia***				
Any	26	38	36	41
None	42	62	52	59

Patients were excluded if they had salivary gland tumours, underwent prior surgery and/or underwent re-irradiation. An additional requirement was that patient-rated follow-up information at 6 and/or 12 months after radiotherapy was available.

CT scans (Somatom Sensation Open, Siemens, Forchheim, Germany; voxel size: 0.94 × 0.94 × 2.0 mm3; 100–140 kV) were acquired within 2 weeks prior to treatment (CT_0_) and weekly during treatment (CT_w1–6_), where CT_w1_ was generally acquired on the day of the first or second fraction. Patients only received intravenous contrast for CT_0_. All scans were acquired with a thermoplastic mask in their radiotherapy treatment position.

All patients provided written informed consent before starting therapy that their data could be used within the department’s research program. The Dutch Medical Research Involving Human Subjects Act is not applicable to data collection as part of routine clinical practice and therefore, the hospital ethics committee granted us a waiver from needing ethical approval for the conduct of studies based on these data. All patients received standard clinical care of adaptive radiotherapy.

### Endpoints

Patient-rated xerostomia scores were collected prospectively on a routine basis; before, weekly during, and subsequently 6 and 12 months after radiotherapy using the EORTC QLQ-H&N35 questionnaire, as part of the standard follow-up programme (SFP) (NCT02435576)^2,18^. The primary endpoint of this study was moderate-to-severe patient-rated xerostomia at 12 months after radiotherapy (Xer_12m_) and the secondary endpoint was moderate-to-severe patient-rated xerostomia at 6 months (Xer_6m_). This corresponds to the 2 highest scores of the 4-point Likert scale (not, a bit, quite a bit, a lot).

### ∆features definitions

Parotid glands were delineated on the CT_0_ and CT_1_ according to the consensus guidelines of Brouwer *et al*.^19^. Delineations were warped from CT_1_ to the weekly CTs using the deformable image registration tool in the treatment planning system RayStation v5.99 (RaySearch Laboratories, Stockholm, Sweden), the warped structures were carefully checked and manually corrected where necessary.

The radiomics features were extracted from the planning and the weekly CTs with Matlab-based (Mathworks, Natick, MA, USA; version R2014a) in-house developed software. The definitions and formulas were in line with the ‘Image biomarker standardisation initiative’^20^. The geometric changes (geometric ∆features) were calculated by subtracting the radiomics features of CT_w2–6_ from those of CT_0_. This resulted in 15 geometric ∆features per weekly CT, that for example represent volume, surface or compactness changes. Intensity features describe first order and histogram characteristics of CT intensities of a parotid gland (e.g. mean or variance). Textural features describe the intensity heterogeneity and were extracted from the grey level co-occurrence matrix (GLCM)^21^, grey level run-length matrix (GLRLM)^22,23^, grey level size-zone matrix (GLSZM)^24^ and neighbourhood grey tone difference matrix (NGTDM)^25^. Contrast enhancement was only used for CT_0_, and not for the weekly CT-scans. Since this can affect the intensity and texture feature values, CT_w1_ (generally acquired before the 2^nd^ radiation fraction) was considered to be the baseline CT. Hence, intensity and texture changes were quantified by calculating the difference between CT_w1_ and the CT_w2–6_. As the intensity and texture features can be influenced by metal artefacts, slices with metal artefacts were deleted and features were calculated on the remaining slices only.

**Figure 3:**
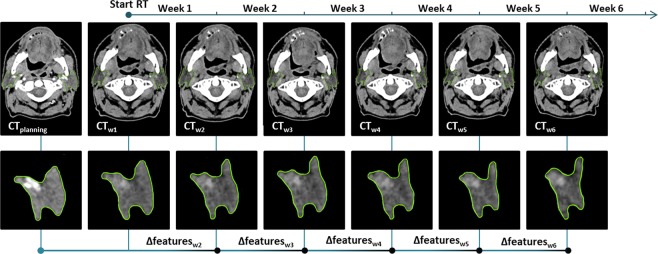
The difference between features extracted from the weekly CTs (CT_w._) and either the planning (geometric features) or week 1 CT (intensity and texture features) resulted in ∆features per week. Generally, scans were taken at the start of consecutive radiation week.

Figure 3 depicts the calculation of the ∆features and CT time points. For a complete list of the 15 geometric, 17 intensity and 66 texture features refer to the Supplementary Datas 1–3, respectively. Only ∆features of the contralateral parotid gland were reported, as they performed better than those of the ipsilateral parotid gland. The geometric ∆features were analysed separately from intensity and texture ∆features.

Clinical variables age and gender were investigated, but not reported as they did not show, to have a significant relation with the development of late xerostomia, which is in line with previous analyses^2,26,27^.

### ∆feature selection

To identify the most predictive ∆features, ∆feature variable selection was performed each week. Firstly, ∆features values were normalised by taking the difference between each value and the average across patients, and then dividing by the standard deviation. Secondly, a pre-selection that was based on inter-variable correlation was performed to reduce the number of variables. If the (Pearson) correlation of two variables was larger than 0.80, only the ∆feature with the highest univariable association with the endpoint was selected. Thirdly, stepwise forward selection was used to select the most important predictors (likelihood-ratio test: p-value < 0.01)^28^. The entire variable selection procedure (normalisation, pre-selection and forward selection) was repeated on 1000 bootstrapped samples (i.e. with replacement), according to the TRIPOD guidelines^29^. The variable selection frequencies were evaluated to identify the most stable predictive variables per week. The Pearson correlation between the selected ∆features was also investigated.

### ∆feature: univariable analysis

In order to identify the optimum week for predicting Xer_12m_ with ∆features, the univariable associations were investigated for the selected ∆features per week in the entire cohort.

### ∆feature, dose and toxicity: multivariable analysis

A reference ‘pre-treatment model’ that was based on baseline xerostomia scores (Xer_baseline_; none vs. any) and the contralateral PGdose, was fitted to the current train set^2^. The prediction performance of the ‘pre-treatment model’ was first compared with that of models based on Xer_baseline_ and the selected ∆features. Subsequently, the addition of the selected ∆features to the ‘pre-treatment model’ was investigated in terms of significance (likelihood-ratio test) and performance.

The resulting multivariable logistic regression models were tested in the unseen data of the test set. Model discrimination was measured with the area under the receiver operating characteristic curve (AUC) and the discrimination slope. Nagelkerke R2 was used as a measure for explained variance. Ultimately, the final model coefficient estimations were performed on the entire cohort. Model calibration was tested for these final models with the Hosmer–Lemeshow test and by repeating the entire variable selection on 1000 bootstrap samples, and by calculating the average of all resulting linear predictor slopes and intercepts. The coefficients were corrected for optimism according to this internal validation procedure.

Since our previous study showed that acute xerostomia scores significantly improved the ∆feature model 6 weeks after treatment^8^, we also investigated if the addition of acute toxicity as assessed during treatment to the ∆feature-models improved model performance.

The relationships between the resulting ∆feature predictors, Xer_baseline_, PGdose and acute xerostomia scores, were additionally explored with univariable logistic regression.

### Parotid gland dose and ∆features

The relationship of the mean contralateral PGdose and the ∆features was investigated with linear regression. Model performance was measured with the coefficient of determination (R2), and normality of the residuals of the regression models was checked.

## Supplementary information

Supplementary Dataset 1–8
