# Supplementary material for: Delta-radiomics features during radiotherapy improve the prediction of late xerostomia
Source: Sci Rep. 2019 Aug 28;9:12483. doi: 10.1038/s41598-019-48184-3 (PMC6713775; doi:10.1038/s41598-019-48184-3)
Supplement: Supplementary file 1 — Supplementary Dataset 1–8 [file 41598_2019_48184_MOESM1_ESM.pdf]

# Supplementary data of

## Delta-radiomics features during radiotherapy improve the prediction of late xerostomia

Lisanne V. van Dijk[a], Johannes A. Langendijk[a], Tian-Tian Zhai[a,b], Thea A. Vedelaar[a], Walter Noordzij[c], Roel J.H.M. Steenbakkers[a], Nanna M. Sijtsema[a]

[a] Department of Radiation Oncology, University of Groningen, University Medical Center Groningen, Groningen, The Netherlands
[b] Department of Radiation Oncology, Cancer Hospital of Shantou University Medical College, Shantou, China
[c]Nuclear Medicine and Molecular Imaging, University of Groningen, University Medical Center Groningen, Groningen, The Netherlands

# Supplementary data 1

### Geometric radiomic features or image biomarkers

| features | Remark | Formula |
|---|---|---|
| Volume | Volume | imVolume* |
| Volume density | Ratio bounding box volume and volume | imVolumeDensity* |
| Volume density ellips | Ratio of ellipsoid enclosing volume and volume | $\frac{3V}{4\pi \cdot a \cdot b \cdot c}$ |
| Surface | Total surface area | imSurface* |
| Surface density | Ratio of surface estimate to volume of the structure | imSurfaceDensity* |
| Surface to volume ratio | | Surface/Volume |
| Compactness1 | A measure of compactness of the structure's shape | $\frac{Volume^2}{\sqrt{\pi}Surface^{\frac{2}{3}}}$ |
| Compactness2 | A measure of compactness of the structure's shape | $36\pi\frac{Volume^2}{Surface^3}$ |
| Sphericity | Indicating deviation of structure from perfect sphere | $\frac{\sqrt[3]{\pi}(6 \cdot Volume)^{\frac{2}{3}}}{Surface}$ |
| Asphericity | Indicating roundness of structure | $\sqrt[3]{\frac{Surface^3}{36\pi \cdot Volume^2}} - 1$ |
| Spherical Disproportion | A measure of surface regularity | $\frac{Surface}{4\pi Radius^2}$ |
| Volume of bounding box | The smallest cubic volume containing the structure | - |
| Volume times mean intensity | - | $\mu \cdot Volume$ |
| Maximum 3D axis | | |
| DistIcent | Distance between centroid and intensity weighted centroid | |

* Geometric measures in by David Legland (2011):

http://www.mathworks.com/matlabcentral/fileexchange/33690-geometric-measures-in-2d-3d-images

## Supplementary data 2:

## intensity features or image biomarkers

*I* CT intensity value per voxel

*N* Number of voxels

*H* The first order histogram with x discrete intensity levels

| features | Remark | Formula |
|---|---|---|
| Mean | Mean intensity value of the structure | $\mu = \frac{\sum I}{N}$ |
| Variance | Variance of intensity values | $\mathrm{VAR} = \sum \lvert I - \mu \rvert^2 / (N-1)$ |
| Minimum | Minimum of intensity values | $\mathrm{Min(I)}$ |
| Maximum | Maximum of intensity values | $\mathrm{Max(I)}$ |
| Range | Range of intensity values | $\mathrm{Max(I)} - \mathrm{Min(I)}$ |
| Root mean square (RMS) | The square root of the mean of the squared intensity values | $\surd \frac{\sum I^2}{N}$ |
| Uniformity | Measure of the uniformity of the histogram | $\sum H^2$ |
| Energy | Amount of information | $\sum I^2$ |
| Skewness | Asymmetry of intensity values around the mean intensity | $\frac{\sum((I-\mu)^3)/N}{\sqrt{\sum((I-\mu)^2)/N}^3}$ |
| Kurtosis | Measure of the tailedness of the histogram | $\frac{\sum((I-\mu)^4)/N}{(\sum((I-\mu)^2)/N)^2}$ |
| $10^{th}$ percentile | Intensity of the $10^{th}$ percentile | P10 |
| $50^{th}$ percentile | Median intensity | - |
| $90^{th}$ percentile | Intensity of the $90^{th}$ percentile | P90 |
| Interquartile range | Range between $3^{rd}$ quartile and $1^{th}$ quartile | $Q_{75}$-$Q_{25}$ |
| Entropy | Measure of randomness | $\sum(H\log_2 H)$ |
| Mean absolute deviance (MAD) | The mean of the absolute deviations of all voxel intensities around the mean intensity value | $\sum \lvert I-\mu \rvert / N$ |
| Robust mean absolute deviance (MAD_rob) | The MAD of the intensities of structure between P10 and P90 | - |

# Supplementary data 3:

# Textural features or image biomarkers

Table. List of textural features

| GLCM features | GLRLM an GLSZM features |
|---|---|
| Autocorrelation (autocorr) | Short Run Emphasis (SRE) |
| Contrast (contr) | Long Run Emphasis (LRE) |
| Haralick's Correlation (corr_Har) | Gray-Level Nonuniformity (GLN) |
| Cluster Prominence (clusprom) | Gray-Level Nonuniformity normalised (GLN_nor) |
| Cluster Shade (clusshade) | Run Length Nonuniformity (RLN) |
| Cluster tendency (clustend) | Run Length Nonuniformity normalised (RLN_nor) |
| Dissimilarity (dissi) | Run Percentage (RP) |
| Energy (Angular second moment) (energ) | Low Gray-Level Run Emphasis (LGRE) |
| Maximum probability (maxpr) | High Gray-Level Run Emphasis (HGRE) |
| Joint average (joint_avg) | Short Run Low Gray-Level Emphasis (SRLGE) |
| Joint variance (joint_var) | Short Run High Gray-Level Emphasis (SRHGE) |
| Joint entropy (joint_ent) | Long Run Low Gray-Level Emphasis (LRLGE) |
| Sum average (sum_avg) | Long Run High Gray-Level Emphasis (LRHGE) |
| Sum variance (sum_var) | Long Run High Gray-Level Emphasis 2 (LRHG2E) |
| Sum entropy (sum_ent) | Long Run High Gray-Level Emphasis 3 (LRHG3E) |
| Difference average (diff_avg) | Grey level variance (GLvar) |
| Difference variance (diff_var) | Run length variance (RLvar) |
| Difference entropy (diff_ent) | Run entropy (RE) |
| Information measure of correlation1 (indnc) | |
| Information measure of correlation2 (idmnc) | **Neighbourhood grey tone features** |
| Inverse variance (inv_var) | Coarseness |
| Inverse difference (homom) | Contrast |
| Inverse difference moment (homomn) | Busyness |
| Inverse difference normalized (inf1h) | Complexity |
| Inverse difference moment normalized (inf2h) | Strength |

For this study specifically, average of features from GLCM and GLRLM in 4 independent directions were used (2D), since slices were metal artefact slices were removed.

For the other formulas of these textural features refer to: Zwanenburg A, Leger S, Vallières M, Löck S. Image biomarker standardisation initiative - feature definitions. arXiv:161207003 2016.

# Supplementary data 4: frequency plots

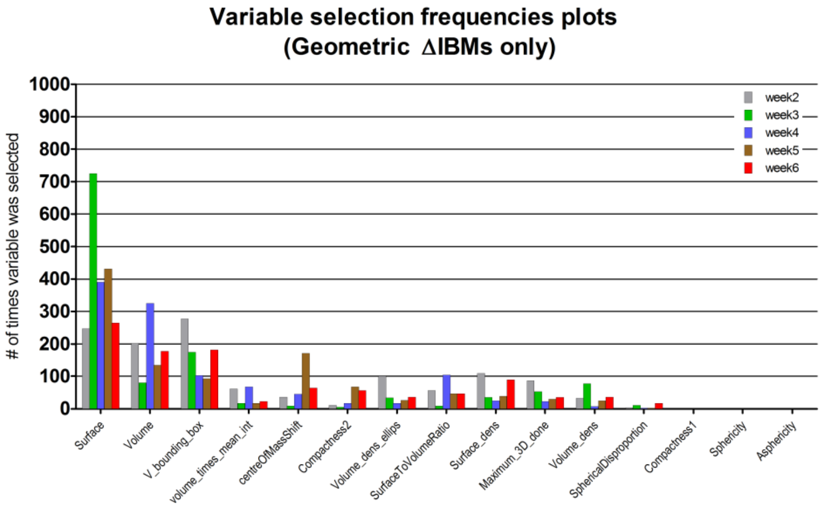


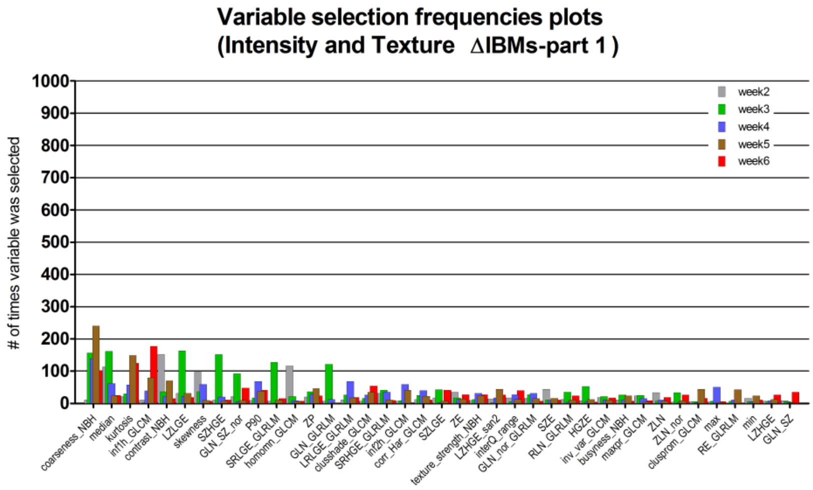


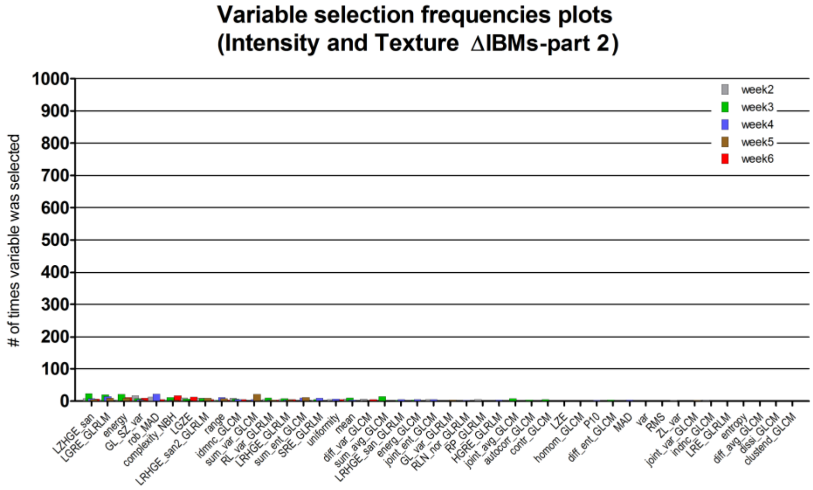

# Supplementary data 5:
# Heatmap of ΔIBMs of week 3

Z-scores were calculated for all ΔIBMs, such that their mean values are 0 (and a standard deviation is 1) and outliner outside the 5th and 95th range were scaled to the 5th and 95th percentiles, respectively. The heatmap was created using the R-package heatmaply (version 0.15.2)[1] .

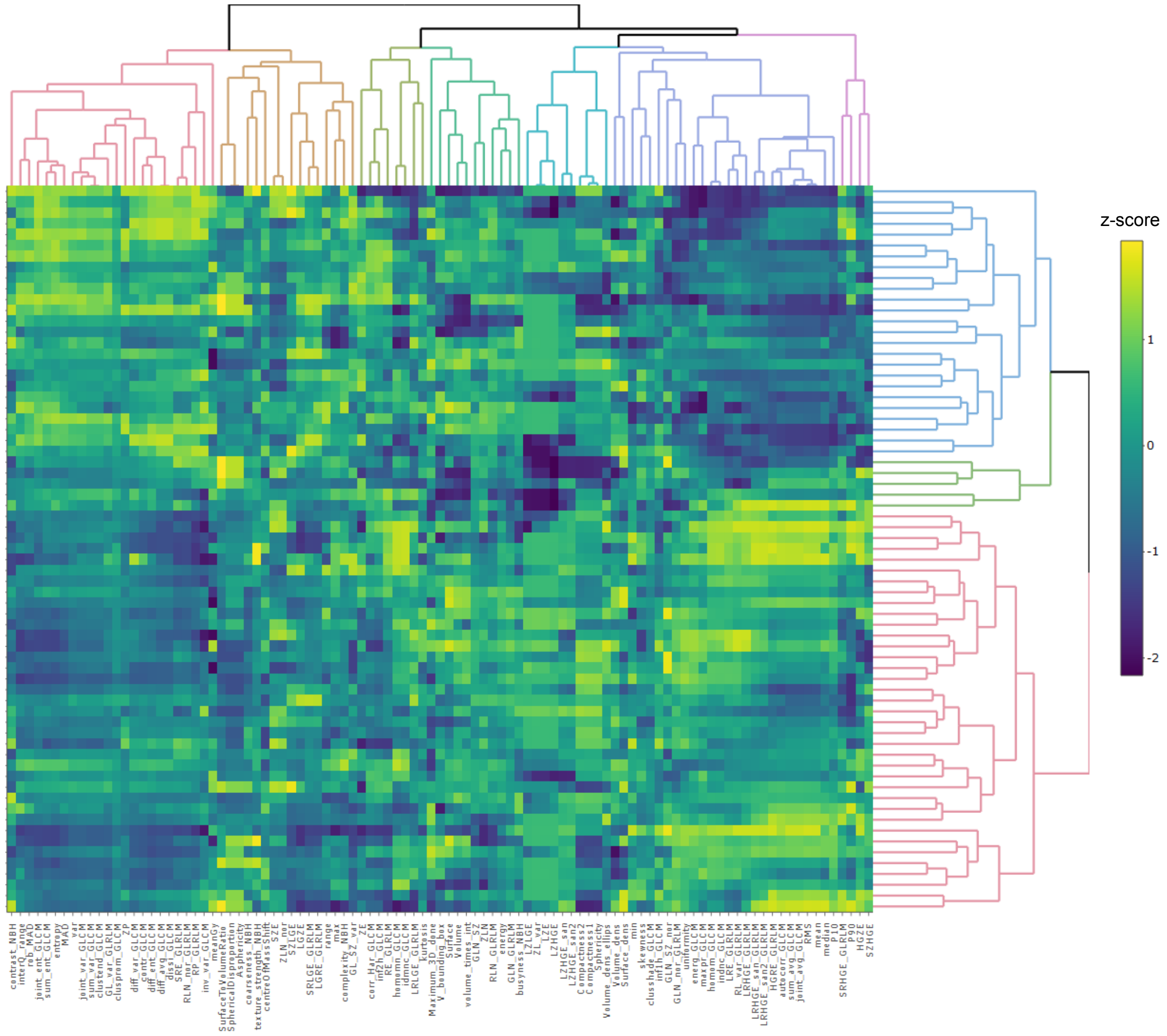


1. Tal Galili, Alan O'Callaghan, Jonathan Sidi, Carson Sievert; heatmaply: an R package for creating interactive cluster heatmaps for online publishing, Bioinformatics, , btx657, https://doi.org/10.1093/bioinformatics/btx657

# Histograms of most frequently selected week 3 ΔIBMs

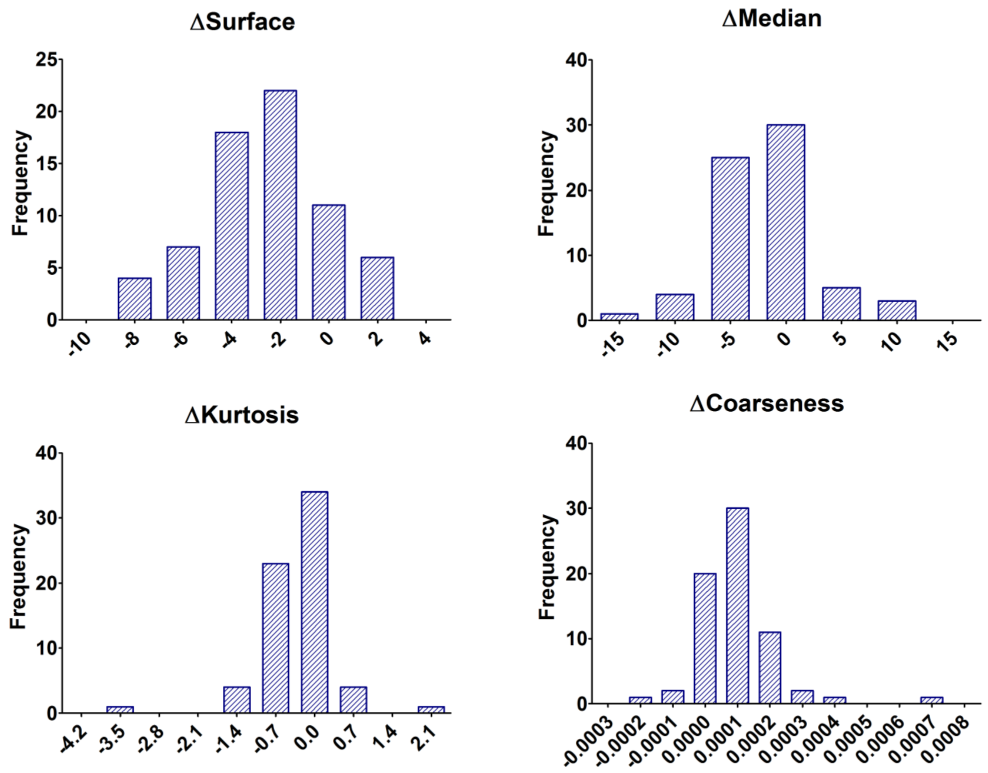


# Histograms of most frequently selected week 3 ΔIBMs (normalised)

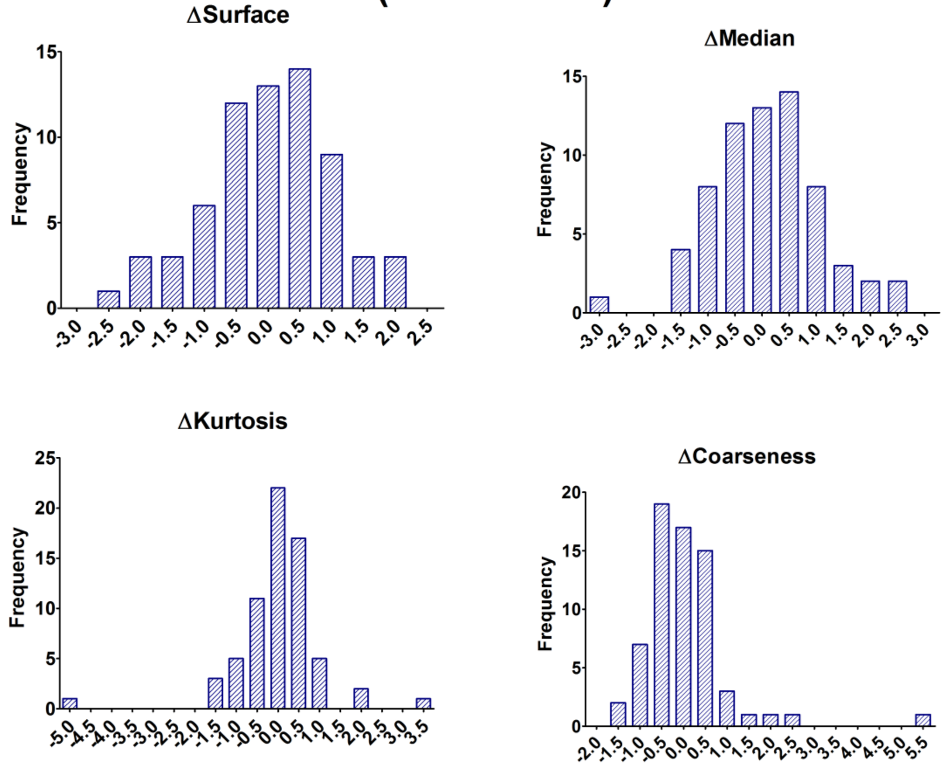

# Supplementary data 6:
# Univariable analysis

Table. Univariable logistic regression analysis of the selected ∆feature and pre-treatment contralateral parotid gland mean dose (PGdose) and baseline scores ($Xer_{baseline}$) per week for predicting moderate-to-severe xerostomia at 12 and 6 months after treatment.

| | | Xerostomia at 12 months | | | | | Xerostomia at 6 months | | | | |
|---|---|---|---|---|---|---|---|---|---|---|---|
| | | β | OR | p | AUC | $R^2$ | β | OR | p | AUC | $R^2$ |
| PGdose | | 0.085 | 1.09 | 0.006 | 0.69 | 0.20 | 0.062 | 1.06 | 0.008 | 0.65 | 0.13 |
| $Xer_{baseline}$ | | 2.258 | 9.56 | <0.001 | 0.75 | 0.33 | 1.889 | 6.61 | <0.001 | 0.71 | 0.24 |
| ΔSurface | week 2 | -0.308 | 0.73 | 0.018 | 0.66 | 0.15 | -0.292 | 0.75 | 0.008 | 0.66 | 0.14 |
| | Week 3 | -0.525 | 0.59 | <0.001 | 0.78 | 0.34 | -0.341 | 0.71 | 0.001 | 0.73 | 0.22 |
| | Week 4 | -0.384 | 0.68 | 0.002 | 0.73 | 0.26 | -0.301 | 0.74 | 0.001 | 0.72 | 0.21 |
| | Week 5 | -0.178 | 0.84 | 0.023 | 0.67 | 0.14 | -0.167 | 0.85 | 0.009 | 0.68 | 0.14 |
| | Week 6 | -0.150 | 0.86 | 0.038 | 0.66 | 0.12 | -0.153 | 0.86 | 0.012 | 0.69 | 0.13 |
| ΔMedian | Week 2 | -0.218 | 0.80 | 0.401 | 0.50 | 0.05 | -0.495 | 0.61 | 0.036 | 0.64 | 0.09 |
| | Week 3 | -0.525 | 0.59 | 0.068 | 0.65 | 0.12 | -0.352 | 0.70 | 0.120 | 0.59 | 0.06 |
| | Week 4 | -0.403 | 0.67 | 0.147 | 0.60 | 0.08 | -0.267 | 0.77 | 0.232 | 0.54 | 0.05 |
| | Week 5 | -0.291 | 0.75 | 0.273 | 0.56 | 0.06 | -0.358 | 0.70 | 0.119 | 0.60 | 0.07 |
| | Week 6 | -0.264 | 0.77 | 0.319 | 0.53 | 0.06 | -0.289 | 0.75 | 0.198 | 0.57 | 0.06 |
| ΔKurtosis | Week 2 | 0.085 | 1.09 | 0.736 | 0.41 | 0.04 | 0.233 | 1.26 | 0.480 | 0.39 | 0.05 |
| | Week 3 | 0.177 | 1.19 | 0.509 | 0.55 | 0.04 | -0.137 | 0.87 | 0.541 | 0.42 | 0.04 |
| | Week 4 | 0.206 | 1.23 | 0.425 | 0.49 | 0.04 | 0.181 | 1.20 | 0.541 | 0.41 | 0.04 |
| | Week 5 | 0.637 | 1.89 | 0.083 | 0.62 | 0.11 | 0.101 | 1.11 | 0.669 | 0.45 | 0.04 |
| | Week 6 | 0.181 | 1.20 | 0.487 | 0.51 | 0.05 | 0.237 | 1.27 | 0.336 | 0.48 | 0.04 |
| ΔCoarseness | Week 2 | 0.122 | 1.13 | 0.625 | 0.51 | 0.04 | -0.082 | 0.92 | 0.708 | 0.36 | 0.04 |
| | Week 3 | 0.619 | 1.86 | 0.062 | 0.68 | 0.12 | 0.494 | 1.64 | 0.071 | 0.65 | 0.09 |
| | Week 4 | 0.371 | 1.45 | 0.160 | 0.65 | 0.08 | 0.216 | 1.24 | 0.328 | 0.58 | 0.05 |
| | Week 5 | 0.435 | 1.55 | 0.102 | 0.61 | 0.08 | 0.432 | 1.54 | 0.062 | 0.62 | 0.08 |
| | Week 6 | 0.360 | 1.43 | 0.168 | 0.57 | 0.07 | 0.485 | 1.62 | 0.041 | 0.63 | 0.09 |

Abbreviations: Median HU intensity; Coarseness of the Neighbourhood Grey Tone Difference matrix; β: regression coefficents; OR: odds ratio; AUC: Area Under the Curve; $R^2$: Nagelkerke $R^2$

# Supplementary data 7:
# Model performance of final model fitted to the entire dataset

Table. Performance of NTCP models predicting $Xer_{12m}$ with and without Δimage biomarkers.
* No variable selection was performed for internal validation of the reference model

| | Pre-treatment reference model | Δfeature model 1 | Δfeature model 2 |
|---|---|---|---|
| | $Xer_{baseline}$<br>PG dose | $Xer_{baseline}$<br><br>ΔPG-Surface $_{w3}$ | $Xer_{baseline}$<br>PG dose<br>ΔPG-Surface $_{w3}$ |
| Nagelkerke $R^2$ | 0.45 | 0.53 | 0.57 |
| Area Under the Curve (AUC) | 0.83 (0.72-0.94) | 0.87 (0.79-0.96) | 0.91 (0.85-0.98) |
| Discrimination slope | 0.37 | 0.43 | 0.47 |
| Hosmer–Lemeshow test $X^2$ (p-value) | 3.96 (p=0.41) | 5.19 (p=0.27) | 5.80 (p=0.21) |
| AUC $_{corrected}$ | 0.81* | 0.84 | 0.87 |
| Nagelkerke $R^2_{corrected}$ | 0.40* | 0.45 | 0.46 |
| Calibration slope (intercept) | 0.90* (-0.03) | 0.82 (-0.06) | 0.75 (-0.07) |

# Supplementary data 8:
# Model characteristics and performance measure predicting xerostomia 6 months after radiotherapy

Table. Estimated coefficients (uncorrected and corrected for optimism) of pre-treatment reference and Δ feature models

| | β | | OR (95% CI) | p-value |
|---|---|---|---|---|
| | Uncorrected | Corrected | | |
| *intercept* | -1.888 | -1.774 | | |
| $Xer_{baseline}$ | 1.856 | 1.744 | 6.40 (2.32-17.65) | <0.001 |
| PGdose | 0.059 | 0.056 | 1.06 (1.01-1.12) | 0.019 |
| *intercept* | -1.404 | -1.340 | | |
| $Xer_{baseline}$ | 1.740 | 1.660 | 5.70 (2.03-15.98) | 0.001 |
| $\Delta PG\text{-}surface_{w3}$ | -0.308 | -0.294 | 0.73 (0.6-0.91) | 0.004 |
| *intercept* | -2.067 | -1.853 | | |
| $Xer_{baseline}$ | 1.758 | 1.574 | 5.80 (2.01-16.72) | 0.001 |
| PGdose | 0.038 | 0.034 | 1.04 (0.98-1.1) | 0.162 |
| $\Delta PG\text{-}surface_{w3}$ | -0.255 | -0.228 | 0.78 (0.62-0.97) | 0.023 |

Abbreviations:

$Xer_{baseline}$: xerostomia at baseline ; PG dose: contralateral mean dose to parotid gland; $\Delta PG\text{-}Surface_{w3}$: Parotid gland surface change from before and week 3 during treatment.

β: regression coefficents; OR: odds ratio; CI: confidence interval

N.b. Surface change in $cm^2$

Table. Performance of reference and Δ feature models

| | $Xer_{baseline}$ PGdose | $Xer_{baseline}$ $\Delta PG\text{-}Surface_{w3}$ | $Xer_{baseline}$ PGdose $\Delta PG\text{-}Surface_{w3}$ |
|---|---|---|---|
| Nagelkerke $R^2$ | 0.30 | 0.34 | 0.37 |
| Area Under the Curve (AUC) | 0.75 (0.64-0.85) | 0.79 (0.70-0.88) | 0.80 (0.74-0.92) |
| Discrimination slope | 0.23 | 0.27 | 0.29 |
| HL test $X^2$ (p-value) | 11.46 (p=0.18) | 5.53 (p=0.70) | 9.38 (p=0.31) |
| $AUC_{corrected}$ | 0.74 | 0.78 | 0.78 |
| Nagelkerke $R^2_{corrected}$ | 0.27 | 0.32 | 0.31 |
| Calibration slope | 0.94 | 0.95 | 0.90 |

Abbreviations: HL: Hosmer–Lemeshow; corrected: corrected for optimism with bootstrapping; $Xer_{baseline}$: xerostomia at baseline; PG dose: contralateral mean dose to parotid gland; $\Delta PG\text{-}Surface_{w3}$: Parotid gland surface change from before and week 3 during treatment.

# Supplementary data 9:
# Relation dose and intensity and texture Δ features

| PGdose ~ Δcoarseness | | | | |
|---|---|---|---|---|
| | β | SE | p | $R^2$ |
| week 2 | -0.001 | 0.01 | 0.941 | 0.00 |
| Week 3 | 0.022 | 0.01 | 0.059 | 0.05 |
| Week 4 | 0.009 | 0.01 | 0.438 | 0.01 |
| Week 5 | 0.043 | 0.01 | 0.000 | 0.21 |
| Week 6 | 0.031 | 0.01 | 0.006 | 0.11 |

| PGdose ~ Δmedian | | | | |
|---|---|---|---|---|
| | β | SE | p | $R^2$ |
| week 2 | -0.023 | 0.01 | 0.047 | 0.06 |
| Week 3 | -0.030 | 0.01 | 0.009 | 0.10 |
| Week 4 | -0.022 | 0.01 | 0.056 | 0.05 |
| Week 5 | -0.032 | 0.01 | 0.004 | 0.12 |
| Week 6 | -0.027 | 0.01 | 0.019 | 0.08 |

| PGdose ~ ΔLZLGE | | | | |
|---|---|---|---|---|
| | β | SE | p | $R^2$ |
| week 2 | -0.013 | 0.01 | 0.266 | 0.02 |
| Week 3 | -0.021 | 0.01 | 0.068 | 0.05 |
| Week 4 | -0.007 | 0.01 | 0.562 | 0.01 |
| Week 5 | -0.018 | 0.01 | 0.122 | 0.04 |
| Week 6 | -0.018 | 0.01 | 0.118 | 0.04 |

## Supplementary data 10:
## Validation of ΔPG-surface models at different time points

Two models trained in independent cohorts validated in the two cohorts, where the same Δfeature, ΔPG-Surface, was measured at different time points: in week 3 during treatment and 6 weeks after treatment. Regression coefficient (of the intercept, $Xer_{baseline}$, parotid gland dose and ΔPG- $surface_{w6-postRT}$) that were obtained (i.e. trained) in cohort A were tested in cohort B with ΔPG-$Surface_{w3}$ and vice versa.

**Cohort A**: ΔPG-Surface extracted from the planning CT and 6 weeks after radiotherapy.

**Cohort B**: ΔPG-Surface extracted from the planning CT and 3 weeks during treatment.

Accordingly, the ΔPG-$surface_{w6-postRT}$ and ΔPG-$surface_{w3}$ models had the following coefficients:

| **Model trained on cohort A** | **β** |
|---|---|
| *intercept* | -2.389 |
| $Xer_{baseline}$ | 1.175 |
| PGdose | 0.022 |
| ΔPG-$surface_{w6-postRT}$ | -0.100 |

| **Model trained on cohort B** | **β** |
|---|---|
| *intercept* | -3.952 |
| $Xer_{baseline}$ | 2.236 |
| PGdose | 0.062 |
| ΔPG-$surface_{w3}$ | -0.415 |

*PG: parotid gland*

The performance of 'model trained on cohort A' (with ΔPG-$surface_{w6-postRT}$) and 'model trained in cohort B' (with ΔPG-$surface_{w3}$) tested in Cohort A ($CT_{w6-postRT}$ –$CT_{w0}$) and Cohort B (Δ$CT_{w3}$ –$CT_{w0}$). In other words, the regression coefficient was used for ΔPG-surface at different time points (e.g. model with *linear predictor = -2.389 + 1.175 * $Xer_{baseline}$ + 0.022 * PGdose + -0.100 ΔPG-$surface_{w3}$* externally validated in Cohort B; final column of Table)

| | **Validation on Cohort A** | | **Validation on Cohort B** | |
|---|---|---|---|---|
| | Model trained on Cohort A | Model trained on Cohort B | Model trained on Cohort B | Model trained on Cohort A |
| Area Under the Curve (AUC) | 0.77 | 0.80 | 0.91 | 0.89 |
| Nagelkerke $R^2$ | 0.38 | 0.32 | 0.57 | 0.53 |

Table. Demographics of patients:

| | **Cohort A** | | **Cohort B** | |
|---|---|---|---|---|
| Characteristics | N=107 | % | N=68 | % |
| *Sex* | | | | |
| Female | 12 | 11 | 20 | 29 |
| Male | 95 | 89 | 48 | 71 |
| *Age* | | | | |
| 18-65 | 67 | 63 | 48 | 71 |
| >65 | 40 | 37 | 20 | 29 |
| *Tumour site* | | | | |
| Oropharynx | 28 | 26 | 22 | 32 |
| Hypopharynx | 7 | 7 | 0 | 0 |
| Nasopharynx | 3 | 3 | 5 | 7 |
| Larynx | 65 | 61 | 22 | 32 |
| Oral cavity | 2 | 2 | 15 | 22 |
| Unknown primary | 2 | 2 | 1 | 1 |

| | | | | |
|---|---|---|---|---|
| Other | - | - | 3 | 4 |
| *Tumour classification* | | | | |
| T0 | 3 | 3 | 1 | 1 |
| T1 | 25 | 23 | 10 | 15 |
| T2 | 47 | 44 | 14 | 21 |
| T3 | 18 | 17 | 17 | 25 |
| T4 | 14 | 13 | 23 | 34 |
| Unknown | - | - | 3 | 4 |
| *Node classification* | | | | |
| N0 | 67 | 63 | 23 | 34 |
| N1 | 8 | 7 | 9 | 13 |
| N2abc | 29 | 27 | 31 | 46 |
| N3 | 3 | 3 | 3 | 4 |
| *Systemic treatment* | | | | |
| Yes | 28 | 26 | 34 | 50 |
| No | 79 | 74 | 34 | 50 |
| *Treatment technique* | | | | |
| 3D-CRT | 22 | 21 | - | - |
| IMRT | 85 | 34 | 27 | 40 |
| VMAT | - | - | 41 | 60 |
| *Bilateral* | | | | |
| Yes | 67 | 63 | 57 | 84 |
| no | 40 | 37 | 11 | 16 |

Abbreviations: IMRT: Intensity-Modulated Radiation Therapy; VMAT: Volumetric Arc Therapy
